# Biochemical diagnosis of acromegaly without a typical clinical phenotype: what are the concerns?

**DOI:** 10.1590/2359-3997000000301

**Published:** 2017-09-01

**Authors:** Antonio Ribeiro-Oliveira, Ariel Barkan

**Affiliations:** 1 Laboratory of Endocrinology Federal University of Minas Gerais Belo Horizonte MG Brazil Laboratory of Endocrinology, Federal University of Minas Gerais (UFMG), Belo Horizonte, MG, Brazil; 2 Department of Medicine Department of Neurosurgery University of Michigan Ann Arbor MI USA Department of Medicine, Division of MEND, and Department of Neurosurgery, University of Michigan, Ann Arbor, MI, USA

The importance of early diagnosis and prompt treatment of acromegaly is beyond question (1). In this issue of *Archives of Endocrinology and Metabolism*, Rosario and Calsolari (2) report the results of a five year follow-up investigation of patients with a suspicious clinical scenario of acromegaly coupled to mildly to modestly elevated IGF-1 but with questionable glucose-suppressed GH. It included 16 women and one man aged 30 to 55 years who were found to have some clinical findings potentially attributable to acromegaly (glucose intolerance, headaches, arthritic pains, etc.) but no acromegalic phenotype. They had circulating IGF-1 levels 1.08 to 1.53 times the upper limit of the normal range for age in two measurements outside puberty or pregnancy, but their glucose-suppressed GH went down below the currently agreed cut-off point of 1 µg/L but was still higher than the strictest criterion of 0.4 µg/L.

All the 17 selected patients had a pituitary MRI performed and non-pituitary acromegaly was excluded through chest and abdominal contrast-enhanced tomography. All of them were not treated for acromegaly (either surgically or medically) but were just followed for up to 5 years and dynamic facial changes was evaluated through longitudinal comparison of photographs. Initially, just one patient had a visible pituitary microadenoma that did not change on follow-up. After five years of follow-up, these patients remained without an acromegalic phenotype and IGF-1 spontaneously returned to normal in 5 out of 17 patients, as confirmed by two measurements. Regarding the other 12 patients with persistently elevated IGF-1, none of them showed an increase of IGF-1 higher than 20%, and 2 of them suppressed GH in oGTT to lower than 0.4 µg/L, while no tumor was further detected. Therefore, after a follow-up of five years, we are still uncertain of the diagnosis in the remaining 10 patients, which means ~60% of the cohort of patients with elevated IGF-1 levels without GH suppression in oGTT to below 0.4 µg/L.

Importantly, in this population without a clinical phenotype of acromegaly, the oGTT cut-off of 1 µg/L, as suggested by the current Endocrine Society consensus (3) would have ended up further investigation at the initial presentation, although high IGF-1 levels would still be without an explanation. The five years follow-up was able to confirm that there was no appearance of facial acromegalic changes or further deterioration of biochemical parameters in these patients. Conversely, over one third of the patients were able to normalize either IGF-1 or reach GH suppression levels < 0.4 µg/L during an oGTT after five years of first assessment. Although quite unlikely, these patients should always be evaluated in the context of genetic syndromes associated with acromegaly, when alteration of biochemical parameters may be subtle (4). Another possibility discussed by the authors, is that these patients may have had slightly elevated IGF-1 level expected in 2.5% of normal population due to statistical distribution of “normalcy”.

The authors suggest that the oGTT criterion of GH < 1 µg/L may be optimal for excluding acromegaly in patients without convincing clinical findings and with only mildly elevated IGF-1. However, the combination of comorbidities of acromegaly found in these patients still raises suspicion that a mild form of the disease might have been present.

First, it relates to purely technical issues. There had recently been a lot of concerns as related to GH and IGF-1 assays (1). For example, we still do not have glucose suppression criteria that are specific for age, gender and body mass index. For the IGF-1, the authors are to be congratulated for using normative parameters derived from a large population (4,350 adults) from the same locality. However, although the authors seem to be aware of the assay overestimation drift with the Siemens IGF-1 assay, the observed normalization of IGF-1 in the subsequent assessment five years later in 30% of patients could be related to the correction of the assay overestimation drift instead of a true normalization.

However, the most important is the realization that the classical diagnostic criteria of acromegaly no longer apply. Recent technical refinements have demonstrated that a significant proportion of patients with acromegaly differ dramatically from “classical” clinical, biochemical and radiological presentations. Indeed, patients with a typical clinical phenotype coupled to high IGF-1 levels, but with plasma GH in the “normal” range may have glucose-suppressed GH below 1 µg/L in ~50% of the cases and below 0.4 µg/L in as many as 30% of the cases (5). Although rare, these cases of “micromegaly” are usually, albeit not necessarily, coupled to the presence of microadenomas (5,6). In these cases when pituitary MRI confirms the presence of the tumor, neurosurgical approach is usually followed by amelioration of the signs and symptoms as well as confirmation of the somatotroph tumor by pathological analysis and immunohistochemistry. Occasionally, cases of acromegaly without a visible tumor have been reported (7). In these cases, the best approach is yet to be defined, although neurosurgical successes have been reported (7). Perhaps, the most difficult is the situation where biochemically and immunochemically – confirmed somatotropinomas are “silent”, i.e. found in patients without any phenotypical manifestations (8,9). In these cases, we are completely in the dark: should we treat the patient or the biochemical/radiological finding?

Holdaway and cols. (10) introduced the “high IGF” parameter as a predictor of higher mortality in acromegaly, but we still do not know exactly how high it should be to have clinical impact on morbidities and mortality justifying active normalization of IGF-1 levels, especially in patients whose clinical diagnosis of acromegaly is not firmly defined.

Cushing disease is famous for being diagnostically the most difficult variety of pituitary tumors. Acromegaly is rapidly gaining on it.
